# Properties Enhancement of High Molecular Weight Polylactide Using Stereocomplex Polylactide as a Nucleating Agent

**DOI:** 10.3390/polym13111725

**Published:** 2021-05-25

**Authors:** Purba Purnama, Muhammad Samsuri, Ihsan Iswaldi

**Affiliations:** 1 School of Applied STEM, Universitas Prasetiya Mulya, Tangerang, Banten 15339, Indonesia; ihsan.iswaldi@prasetiyamulya.ac.id; 2 Chemical Engineering Department, Universitas Bhayangkara Jakarta Raya, Bekasi 17121, Indonesia; msamsuri79@gmail.com

**Keywords:** polylactide, stereocomplex, nucleating agent, biopolymer, polymer blend

## Abstract

As one of the most attractive biopolymers nowadays in terms of their sustainability, degradability, and material tune-ability, the improvement of polylactide (PLA) homopolymer properties by studying the utilization of stereocomplex polylactide (s-PLA) effectively and efficiently is needed. In this sense, we have studied the utilization of s-PLA compared to poly D-lactide (PDLA) homopolymers as a nucleating agent for PLA homopolymers. The mechanical and thermal properties and crystallization behavior of PLA homopolymers in the presence of nucleating agents have been evaluated using a universal testing machine, differential scanning calorimeter, and X-ray diffractometer instruments, respectively. PDLA and s-PLA materials can be used to increase the thermal and mechanical properties of poly L-lactide (PLLA) homopolymers. The s-PLA materials increased the mechanical properties by increasing crystallinity of the PLLA homopolymers. PLLA/s-PLA enhanced mechanical properties to a certain level (5% s-PLA content), then decreased them due to higher s-PLA materials affecting the brittleness of the blends. PDLA homopolymers increased mechanical properties by forming stereocomplex PLA with PLLA homopolymers. Non-isothermal and isothermal evaluation showed that s-PLA materials were more effective at enhancing PLLA homopolymer properties through nucleating agent mechanism.

## 1. Introduction

The development of future materials is focused on their sustainability, degradability, and material tune-ability. Sustainability is an important parameter to ensure material resources will have a long life. Material tune-ability is a specific characteristic, which refers to the ability to alter and adjust the properties of the material to suit existing applications. Degradability is related to the environmental issues caused by material waste. Environmental problems receive more attention to recycle or develop the non-degradable material. Accumulation of non-degradable waste from the high consumption of fossil fuels-based materials in various applications causes tremendous environmental problems. Recently, many researchers have focused on the development and modification of the physical–mechanical properties of biodegradable polymers to substitute these for traditional polymers.

Polylactide (PLA) is one of the most attractive biopolymers with many advantages that comply with future material development, such as sustainability, biodegradability, biocompatibility, and properties modification. PLA is a bio-based polymer that is commercially available and has accomplished large-scale production since 2001 [1,2]. It has the potential to replace fossil-based polymers due to its biodegradability and biocompatibility [3,4]. PLA is a stiff and brittle material at room temperature with a glass transition temperature of ~55 °C and a melting temperature of 180 °C. It has weaknesses such as a low crystallization rate and heat distortion temperature, as well as insufficient crystallization ability during common industrial processes. As a thermoplastic biopolymer, it can be amorphous or semicrystalline in nature depending on its enantiomeric structures: Poly L-lactide (PLLA), Poly D-lactide (PDLA), or Poly DL-lactide (PDLLA).

Commercial PLA made from L-lactide is a brittle material. The limitation in its thermal and mechanical stability restricts its wide application to replace oil-based polymers that require high impact strength [5]. Industrial processing of PLA results in a low heat distortion temperature due to its low crystallization rate and degree of crystallinity in a short processing time [6,7]. Considering its wide potential market, enhancement of the properties of PLA is mandatory in order to comply with specific applications. Blending PLA with other materials has been explored for the specific enhancement of PLA properties. The addition of plasticizers into the PLA matrix can reduce its brittleness and enhance its life span. Mixing PLLA and PDLA enantiomers forma stereocomplex PLA (s-PLA) with a different crystal structure and higher thermal and mechanical properties [8,9,10]. Furthermore, the blending of PLA with nucleating agents could improve its thermal and mechanical properties as well as increasing its crystallinity.

As the crystal structure of s-PLA has unique characteristics with higher thermal and mechanical properties, crystalline s-PLA can be utilized as a nucleating agent to improve the thermal and mechanical properties of PLA [8,11]. Many studies have reported strategies to improve the properties of PLA through s-PLA formation [12,13,14,15,16,17].

Rahman et al. reported that the addition of PDLA into PLLA accelerated the crystallization of PLLA homopolymer through enhancement of the nucleation process, but slightly interfered with the crystallization growth [12]. The equimolar addition of high molecular weight PDLA (Mw > 2 × 10^5^ g/mol) into PLLA homopolymer above melting temperature preferably crystallizes to form s-PLA [13]. Ji et al. reported the increasing crystallization rate and nucleation site of PLLA and PDLA homopolymer in the presence of low molecular weight s-PLA [14]. The presence of poly DL-lactide copolymer in the PLLA and PDLA mixture inhibited PDLA chain diffusion during the crystallization process of s-PLA formation [15]. The presence of s-PLA crystalline with various chain structures in the PLA brings higher mechanical and thermal properties [16]. Property enhancement by s-PLA will affect PLA processing: thermal processing, additive manufacturing, and solution casting [17]. Thermal and mechanical enhancement of PLA through stereocomplexation has been utilized to obtain stable PLA materials which are suitable for many applications, especially use as high-performance materials [8,14,15,16,17,18,19,20,21]. The mechanical and thermal properties of s-PLA are higher than those of pure PLA films, which is caused by the strong molecular interactions (hydrogen bonds and dipole-dipole interactions) of PLLA and PDLA chains [21,22,23,24].

The stereocomplexation of PLA is a very well-known strategy for enhancing the properties of PLA-based materials by blending PLLA and PDLA homopolymers. But, it is requires an effective s-PLA production method. Previously, we have developed the effective stereocomplexation of high-molecular-weight PLA through supercritical fluid technology [24,25,26]. s-PLA also has some constraints for its use in real applications due to its melt stability during thermal processing in industries and also the high cost of PDLA in equimolar ratio with PLLA. Nevertheless, with its crystalline structure and characteristics, s-PLA materials can be used as a nucleating agent. Additionally, the material form of s-PLA is important to comply with industrial processing. By using supercritical fluid technology, a perfect s-PLA is obtained in a dry, powder form which is suitable to be used as a nucleating agent [24]. Utilization of s-PLA materials as a nucleating agent (additives) instead of PDLA homopolymer will reduce the use of PDLA by up to a half. As commercial applications require competitive costs, the low consumption of PDLA homopolymer will be beneficial for reducing production costs.

For these reasons, it is necessary to find an effective and efficient utilization of s-PLA to improve PLA homopolymer properties. In this work, we have studied the utilization of s-PLA compared to PDLA homopolymers as a nucleating agent for PLA homopolymers. We have evaluated the mechanical, thermal properties, and crystallization behavior of PLA homopolymers in the presence of nucleating agents.

## 2. Materials and Methods

### 2.1. Materials

L-Lactide (L-LA) and D-Lactide (D-LA) were purchased from Corbion (Amsterdam, The Netherlands). Tin(II)bis(2-ethylhexanoate) (Sn(Oct)_2_) (Sigma Chemical Co., St. Louis, MO, USA, purity ≥ 99%) and 1-dodecanol (DoOH) (Sigma-Aldrich, St. Louis, MO, USA, purity ≥ 99.5%) were purified by distillation under reduced pressure and dissolved in dry toluene. The toluene was dried by refluxing over a benzophenone-Na complex and distilled in a nitrogen atmosphere just prior to use. High molecular weight PLLA (*M_n_* = 181,493 g/mol, *M_w_* = 425,561 g/mol, PDI = 2.345) was synthesized by ring-opening polymerization of L-lactide (Corbion, Amsterdam, The Netherlands) at 130 °C for 24 h. Other PLA homopolymers for nucleating agent materials: PDLA (*M_n_* = ~87,000 g/mol, *M_w_* = ~125,000 g/mol, PDI = 1.437) and PLLA (*M_n_* = ~87,000 g/mol, *M_w_* = 153,000 g/mol, PDI = 1.759) were also synthesized by a similar method. Dichloromethane (JT Baker, HPLC grade) and CO_2_ (Purity ≥ 99.9%) were used as received.

### 2.2. Stereocomplex Formation

The s-PLA was synthesized by combining PDLA (*M_n_* = ~87,000 g/mol, *M_w_* = ~125,000 g/mol, PDI = 1.437) and PLLA (*M_n_* = ~87,000 g/mol, *M_w_* = 153,000 g/mol, PDI = 1.759) with 1:1 weight ratio and processed through supercritical carbon dioxide–dichloromethane [24]. The processing condition was optimized at 65 °C to achieve a pressure of 350 bar and allowed to proceed for the predetermined times (5 h). The reactor was opened immediately after the reaction had finished obtaining a dry and powder-shaped s-PLA.

### 2.3. Polylactide Blending

S-PLA and D-lactide were prepared as nucleating agents for high molecular weight PLLA materials. PLA blends were prepared by adding s-PLA particles or PDLA homopolymer with various contents into PLLA materials by a solution casting method. We denoted these as PLLA/PDLAx and PLLA/s-PLAx for the blends containing PDLA and s-PLA, respectively. The x values represent the PDLA or s-PLA content in the blends. PLLA/s-PLA3, PLLA/s-PLA5, and PLLA/s-PLA10 represent the PLA blends with 3%, 5%, and 10% of s-PLA particle content, respectively. In a similar notation, PLLA/PDLA3, PLLA/PDLA5, and PLLA/PDLA10 represent the PLA blends with 3%, 5%, and 10% of PDLA content, respectively. Neat PLLA homopolymers were used as control materials. The mixture was dissolved in dichloromethane with total polymer to total solvent ratio (weight to volume) of approximately 5:100. The mixture was vigorously stirred for 4 h and poured into a petri glass. It then underwent evaporation at room temperature for 24 h and was subsequently placed in a vacuum condition at 80 °C for 48 h.

### 2.4. Characterization

The PLA blend films were characterized to evaluate the enhancement of their mechanical and thermal properties. The mechanical testing method is adopted from ASTM D-638 to evaluate tensile properties. The mechanical properties of the PLA blends were measured by Universal Testing Machine (6800 Series, Instron, Norwood, MA, USA) apparatus with a specimen size of 20 mm × 5 mm and sample thickness of approximately 80 μm. The distance between the supports was 10 mm and the extension rate was 1 mm/min. The thermal properties of PLA blends were evaluated using a modulated differential scanning calorimeter (Modulated DSC 2910, TA Instrument, New Castle, DE, USA). The heating rate was fixed at 10 °C/min. Isothermal and non-isothermal crystallizations were evaluated by varying cooling rates and crystallization temperature, respectively. X-ray diffraction spectra were registered with an X-ray diffractometer D/Max-2500 (Rigaku, Japan) composed of Cu *K_α_* (λ = 1.54056 Ǻ, 30 kV, 100 mA) source, a quartz monochromator, and a goniometric plate. A polarized optical microscope was also used to evaluate crystal growth during the isothermal and non-isothermal crystallization processes.

## 3. Results and Discussions

The s-PLA and PDLA we investigated are nucleating agents for PLLA homopolymers. Various reports have studied the nucleating effect of s-PLA by combining PDLA into PLLA homopolymer. Here, we report the use of real s-PLA materials to evaluate the nucleating effect of PLLA homopolymers compared to PDLA as a nucleating agent.

**Figure 1:**
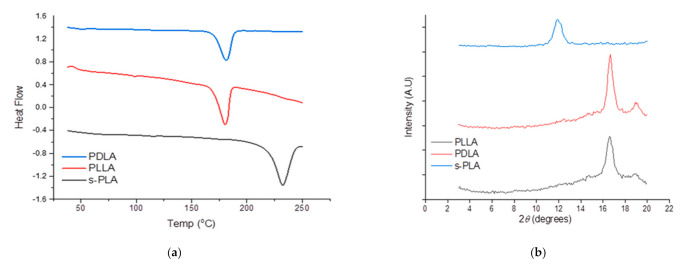
Materials (PLLA, PDLA, and s-PLA) characteristic comparison: (**a**). DSC thermogram; (**b**). X-ray diffraction patterns.

The use of s-PLA as a nucleating agent was successfully synthesized through supercritical carbon dioxide–dichloromethane [24]. The s-PLA and PDLA were evaluated by DSC and XRD instruments to confirm the characteristics of the materials as shown in Figure 1. The synthesized s-PLA crystalline shows a single peak at ~12° of 2*θ* compared with PLLA and PDLA at ~17° and 19° of 2*θ* which indicates the change of crystal structure and helical conformation of PLLA and PDLA driven by hydrogen bonding interactions. The different diffraction peaks mean that all of the PLLA and PDLA blends successfully formed s-PLA [20]. The s-PLA formation was also confirmed by its single melting temperature (*T_m_*) at 230 °C, which was 50 °C higher than *T_m_* of homopolymers (~180 °C).

The s-PLA can be produced by solvent casting [11,12,13,14,15,21,27], thermal processing [28,29], microwave irradiation [30], and the supercritical fluid technology method [24,25,26]. The solvent casting and thermal processing methods have molecular weight constraints to generate perfect s-PLA materials [21,28]. The microwave irradiation method is a fast and efficient process to produce bulk s-PLA materials in a short time [30]. However, it has limitations in producing s-PLA on a large scale. In this work, we obtained s-PLA using supercritical carbon dioxide–dichloromethane. The supercritical carbon dioxide–dichloromethane generated perfect dry and powder-shaped s-PLA materials [24]. It is also possible to scale up this process into commercial production. Furthermore, a dry and powder-shaped s-PLA material is suitable for use as additives in industrial application.

The addition of nucleating agent into the polymer matrix was adopted to improve or enhance specific matrix properties. Improvement of the properties of PLA homopolymers is important in replacing conventional polymeric materials. The addition of s-PLA or PDLA as the nucleating agent was expected to improve the mechanical properties of PLLA homopolymer. The nucleating agent’s amounts in the PLLA homopolymer were varied at 3%, 5%, and 10% weight ratios. The PLLA mechanical properties enhancement with the addition of s-PLA and PDLA are tabulated in Table 1.

The addition of s-PLA or PDLA improves the mechanical properties of PLLA homopolymer. The Young’s modulus of PLLA homopolymer with s-PLA content of 3%, 5%, and 10% increased by 29.90%, 41.74%, and 44.47%, respectively. The s-PLA additions also increased tensile strength up to the addition of 5% s-PLA, then decreased at the addition of 10% of s-PLA. The s-PLA slightly reduced PLLA homopolymer elongation at 3% and 5%, but this drastically reduced at 10% of s-PLA contents. On the other hand, the addition of PDLA only increased Young’s modulus of PLLA by 17.66% at the same content. It also slightly increased tensile strength by 11.21% but increased elongation at break.

Previous studies generally focus on the enhancement of PLA properties through the formation of s-PLA materials from PLLA and PDLA with various blending ratios [9,10,11,12,13,14,15,27]. The mechanical properties of s-PLA increase with an equivalent ratio of PLLA and PDLA, such as Young’s modulus by up to 25% [24]. The enhancement of mechanical properties also depends on the ratio of PLLA to PDLA [14,28]. In general, the mechanical properties of PLA blends improve by up to 25% (Young’s modulus) with 50% of PDLA, then decrease when increasing the PDLA portion [31]. In this work, the addition of s-PLA and PDLA as nucleating agents increased the tensile strength and Young’s modulus of PLA material. Overall, s-PLA and PDLA nucleating agents improve mechanical properties. Moreover, s-PLA makes significant improvements in tensile strength and Young’s modulus compared with PDLA material. On the other hand, s-PLA slightly reduces elongation at break compared to PDLA. When compared to previous research, the addition of a small number of s-PLA materials into a PLA homopolymer contributes significant improvements in tensile strength and Young’s modulus with a slight reduction in elongation at break.

From the data, the addition of s-PLA and PDLA enhanced mechanical properties in a different pattern. The addition of s-PLA is predicted to enhance mechanical properties through a nucleating effect; however, the addition of PDLA enhanced the mechanical properties through the formation of stereocomplex crystallites and acted as an intermolecular cross-link connecting homopolymer crystallites [21].

We also evaluated the thermal properties that were affected by the addition of s-PLA particles and PDLA homopolymers. Based on DSC scanning, the addition of s-PLA enhanced the crystallinity of homopolymers. As shown in Table 2, PLLA homopolymer showed a single melting point (*T_m_*) at ~180 °C, and PLLA blends showed two *T_m_* values (*T_m_*^1^ = ~180 °C and *T_m_*^2^ = ~230 °C). The *T_m_*^1^ is the melting point of PLLA homopolymer crystallites and *T_m_*^2^ is the melting point of s-PLA crystallites. The heat of melting at high temperature (∆*H*^2^) evaluation showed that PDLA has a bigger enthalpy value compared to s-PLA. Theoretically, the same addition of PDLA resulted in approximately double ∆*H*^2^ compared with s-PLA due to the PDLA homopolymer percentage being approximately 50% in s-PLA crystallites. The degree of crystallinity of PLLA/PDLA blends showed a decrease in PLLA crystallites due to some portion of PLLA homopolymers being converted into s-PLA crystallites caused by the hydrogen bonding (CH_3_∙∙∙O=C interaction) between PLLA and PDLA homopolymers [8]. The s-PLA particle slightly increased the degree of crystallinity of PLLA homopolymer crystallites. The presence of s-PLA particles in the blend was confirmed by the *T_m_*^2^ value. The degree of crystallinity (*χ*) of s-PLA from the PLLA/PDLA3 blend showed double the value when compared with the PLLA/s-PLA3 blend. Thus, the thermal evaluation data complies with the theoretical calculation of *T_m_* and ∆*H*^2^.

**Figure 2:**
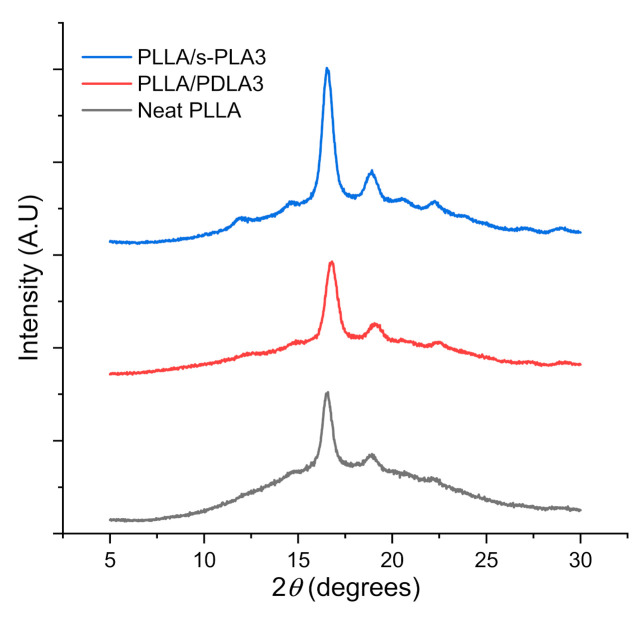
X-ray diffraction pattern of PLLA homopolymer, PLLA/PDLA3 and PLLA/s-PLA blends.

The thermal evaluation data from DSC scanning was confirmed by XRD evaluation as shown in Figure 2. The XRD pattern confirmed the presence of PLLA homopolymer and s-PLA crystallites in the polymer blends. The XRD analysis indicated diffraction peak at 2*θ* = 14.6°, 17°, 19°, 23.7° on homopolymer, whereas diffraction peaks for stereocomplex PLA were observed at 12.5°, 21°, and 24°. X-ray diffraction evaluation confirmed the presence of s-PLA crystallites on PLLA/PDLA3 and PLLA/s-PLA3 blends. The area of diffraction peak of PLLA/s-PLA3 was wider than PLLA/PDLA3 blends. This data aligned with DSC evaluation on the degree of crystallinity. The higher crystallinity will affect the flexibility of the material. Higher crystallinity caused materials to be more rigid and brittle which caused decreases in elongation at the break during mechanical testing.

**Figure 3:**
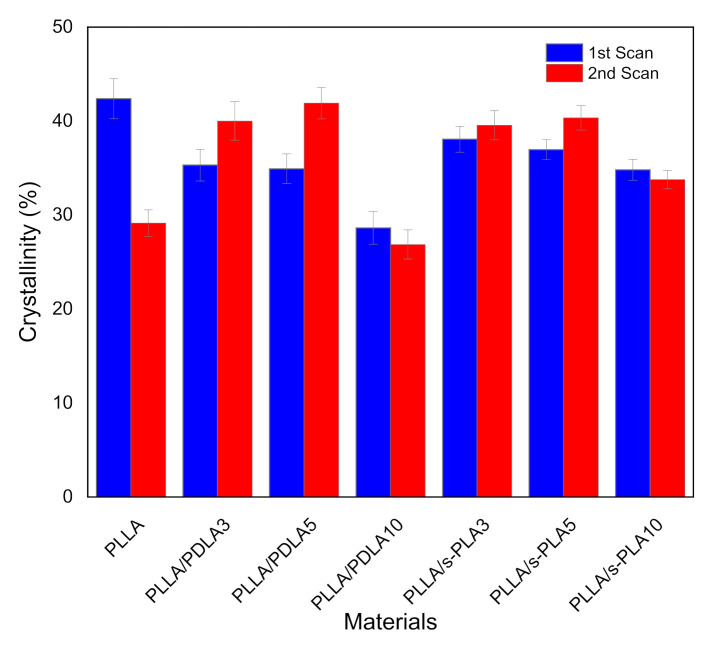
DSC thermogram of first and second scan for PLLA homopolymer, PLLA/PDLA blends, and PLLA/s-PLA blends at scanning rate 10 °C/min.

We also evaluated the melt stability characteristics, or the crystallization process after melting of the blends to ensure their suitability in real industrial processes and applications due to the importance of maintaining the physical properties and processability of the materials. The evaluation on melt stability of the blends was performed by a DSC comparing the degree of crystallinity before and after melting at 250 °C by scanning speed at 10 °C/min (shown in Figure 3). The degree of crystallinity of PLLA homopolymer drastically decreases. The degree of crystallinity of PLLA/PDLA blends shows significant improvement in crystallization after melting, but with a high content of PDLA (10%), the degree of crystallinity drastically decreases in the first and second scanning. Decreases in the degree of crystallinity with high content of PDLA homopolymers are caused by the limitations of high molecular weight PDLA to form stereocomplex crystallites in solution casting (first scan) and to re-assemble the enantiomeric homopolymer chains after melting (second scan) [28]. For the PLLA/s-PLA blends, the degree of crystallinity at the first and second scanning does not have a significant difference compared to PLLA/PDLA blends. From these data, the nucleating effect of s-PLA is more effective compared with PDLA materials due to PDLA materials forming as s-PLA first, then acting as a nucleating agent.

**Figure 4:**
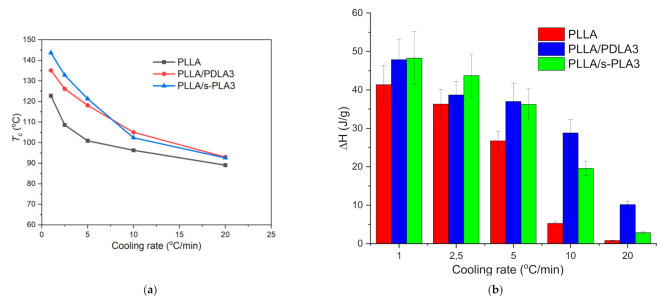
(**a**). Change of cold crystallization temperature (*T*_c_); (**b**). Heat of melting at different scan rates for PLLA, PLLA/PDL3, and PLLA/s-PLA3.

Some studies reported the crystallization behavior of PLA homopolymers in the presence of PDLA as a source of s-PLA crystallites [14,27,29]. Here, we also evaluated the non-isothermal and isothermal crystallization behavior of PLLA homopolymers in the presence of PDLA and s-PLA. For non-isothermal crystallization, the materials were heated to 200 °C at the rate of 10 °C/min and held for 3 min at the same temperature. Based on the non-isothermal crystallization process at different cooling and scanning rates, increasing the scanning rate will decrease the cold crystallization temperature and the heat of melting values as shown in Figure 4. PLLA homopolymers required a certain time to initialize the crystallization process. Aligned with previous reports [27,29], the cold crystallization temperature (*T*_c_) was increased by a slower cooling rate and the presence of a nucleating agent. The presence of s-PLA crystallites in PLLA homopolymers increased *T*_c_ values which correspond to the acceleration of PLLA crystallization [27]. Figure 4 shows the *T*_c_ and ∆*H* values of PLLA/s-PLA3 at a slow cooling rate are higher when compared with PLLA/PDLA3 which means the nucleating effect of s-PLA will show more significant effects compared with PDLA homopolymers. PDLA homopolymers are probably required to form s-PLA before acting as a nucleating agent.

The crystallization study is important to evaluate the material’s behavior during thermal processing. The use of s-PLA as a nucleating agent offers higher effectivity compared with PDLA homopolymer due to PDLA needing to form s-PLA before acting as a nucleating agent. For s-PLA formation during PLLA and PDLA blending, molecular weight and structure is important in structure re-arrangement during the melt. The s-PLA material can act directly as a nucleating agent during the thermal or melt process.

**Figure 5:**
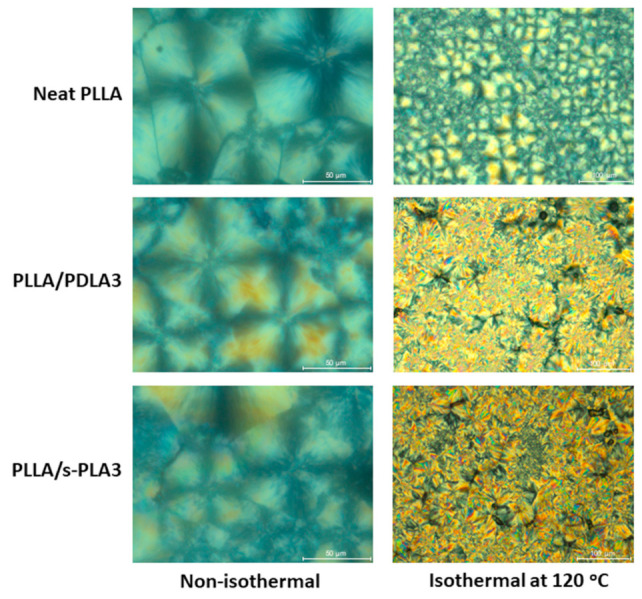
Polarized optical micrographs of PLLA, PLLA/PDLA3, and PLLA/s-PLA3 crystallized through non-isothermal and isothermal crystallization at 120 °C.

To obtain information about crystallization behavior, the polarized optical microscope was used to evaluate the crystal growth of polymeric materials as shown in Figure 5. Non-isothermal crystallization showed that PLLA materials are able to form crystal structures in the presence or absence of a nucleating agent. The crystal formation shows the different size and number of spherulite per area. PLLA homopolymers show a bigger crystal size compared with PLLA/PDLA3 and PLLA/s-PLA3. It means PLLA homopolymer has a slower initialization of crystal formation. PLLA/s-PLA showed a smaller average crystal size compared with PLLA/PDLA3. Therefore, the s-PLA materials have a faster nucleating effect compared with PDLA materials which should form s-PLA crystallites first before acting as a nucleating agent. At isothermal temperature 120 °C, PLLA/s-PLA3 and PLLA/PDLA3 showed a higher density of crystallites compared with PLLA homopolymer. The PLLA/s-PLA3 showed smaller crystallite size and higher density compared with PLLA/PDLA3. Based on these data, s-PLA showed a better nucleating agent compared with PDLA homopolymers.

## 4. Conclusions

The PDLA and s-PLA materials can be used to increase the thermal and mechanical properties of PLLA homopolymers. S-PLA materials enhanced mechanical properties by increasing the crystallinity of the PLLA homopolymers. PLLA/s-PLA enhanced mechanical properties up to a certain level (5% s-PLA content), which then decreased due to a higher amount of s-PLA materials affecting the brittleness of the blends. The addition of s-PLA improved mechanical properties by more than 25% of tensile strength and Young’s modulus. PDLA homopolymers increased mechanical properties by forming stereocomplex PLA with PLLA homopolymers. The addition of 10% PDLA homopolymer improved PLLA homopolymer by up to 11% of tensile strength and 17% of Young’s modulus. Higher content of PDLA homopolymer has difficulties forming perfect stereocomplexation of PLA due to its limitations caused by molecular weight. Non-isothermal and isothermal evaluation showed that s-PLA materials are more effective in enhancing PLLA homopolymer properties through nucleating agent mechanism.

## Figures and Tables

**Table 1:** The mechanical properties of PLLA homopolymers with addition of s-PLA and PDLA as the nucleating agent.

Materials	Elongation at Break (%)	Tensile Strength (MPa)	Young’s Modulus (GPa)
Neat PLLA	5.10 ± 0.26	86.26 ± 4.31	2.27 ± 0.10
PLLA/s-PLA3	4.97 ± 0.20	108.22 ± 5.19	2.95 ± 0.12
PLLA/s-PLA5	4.65 ± 0.17	115.22 ± 5.42	3.22 ± 0.14
PLLA/s-PLA10	3.83 ± 0.11	98.33 ± 3.44	3.28 ± 0.15
PLLA/PDLA3	5.22 ± 0.26	94.86 ± 2.94	2.47 ± 0.10
PLLA/PDLA5	5.29 ± 0.24	94.21 ± 2.73	2.61 ± 0.09
PLLA/PDLA10	5.43 ± 0.22	95.93 ± 4.03	2.67 ± 0.10

**Table 2:** Thermal properties evaluation by Differential Scanning Calorimeter with scanning speed 10 °C/min.

Materials	*T*_m_^1^	∆*H*^1^	*T*_m_^2^	∆*H*^2^	χ^1^	χ^2^	*Xtotal*
Neat PLLA	178.10	37.83	-	-	41.12	-	41.12
PLLA/s-PLA3	178.02	34.17	222.30	4.693	37.14	3.30	40.44
PLLA/PDLA3	179.97	37.87	227.66	2.300	41.16	1.62	42.78

## Data Availability

Data is contained within the article.
